# Coping With Students’ Stress and Burnout: Learners’ Ambiguity of Tolerance

**DOI:** 10.3389/fpsyg.2022.842113

**Published:** 2022-02-17

**Authors:** Jian Xu, Ying Ba

**Affiliations:** ^1^School of Marxism, Dalian Maritime University, Dalian, China; ^2^School of Marxism, Dalian University of Technology, Dalian, China

**Keywords:** learners’ academic stress, ambiguity of tolerance, burnout, positive psychology, emotional fatigue

## Abstract

In the learning milieu, academic stress is deemed as the most general mental condition that learners encounter throughout their educational process, and it has been viewed as one of the most central issues not only in general education but also specifically in language learning. Likewise, burnout has been the main point in this situation. The comprehensive sources of stress and the reasons for burnout are pinpointed in the literature so realizing their association with other aspects such as coping strategies, namely tolerance of uncertainty, are at the center of attention as it may help reduce burnout and decrease the level of stress. To this end, the goal of the present study is to prove the influence of the tolerance of ambiguity in explaining the role of stress and burnout. Briefly, some implications are set forth for the educational stakeholders.

## Introduction

Taking into consideration today’s constantly altering cultural, social, economic, and political climate, it is expected that learners encounter high rates of stress and disclose feelings of being overwhelmed and fatigued (Cheng et al., 2019). Even more important, scholastic stress is encountered because of substantial course work, hard and recurrent testing processes, high self-expectations, and the force to conduct and achieve (Bedewy and Gabriel, 2015). Learners normally encounter difficulties that make the educational setting increasingly stressful when studying a new language, as numerous inquiries have shown, most learners encounter high degrees of academic stress (Choi et al., 2019; Rajoo et al., 2019). In the educational cycle, learners with course stress, high course load, or other mental elements have low emotional exhaustion, and a low individual sense of success (Yang, 2004).

In addition, dropout problems are primarily related to high scholastic stress, as they influence learners in several ways (Kamtsios and Karagiannopoulou, 2015). The extent of stress that learners encounter in the foreign language educational cycle might have harmful influences on their scholastic achievement and success (Iannello et al., 2017). Indeed, stress is a significant element that can influence learners’ education and success as learners studying English may encounter more language stress due to the number of mental, societal, and literary elements (Khattak et al., 2011) and accordingly, burnout is also more popular among EFL learners than other learners that are the result of prolonged chronic stress that bring about issues like emotional fatigue, irritability, and physical symptoms like headaches, and intellectual impairment like memory and concentration issues (Li, 2020), and this construct is associated with a variety of adverse consequences, including poor performance, mental distress, and even dropouts from school (Bask and Salmela-Aro, 2013; Wang and Guan, 2020).

Although there is much literature on educators’ burnout in educational research, learners’ burnout has not been taken into consideration (Robins et al., 2018) and it has become clear, nonetheless, that burnout is not limited to educators; rather, it can correspondingly happen to learners in foreign language settings (Li, 2020). In fact, from a psychological point of view, like any other career, learners are emotionally and behaviorally involved in a variety of activities that may be considered as “work” that these activities are highly structured, mandatory (participating in class, doing homework, and taking examinations), and targeting success goals (Schaufeli and Taris, 2005). Learners’ burnout is a significant element that has a profound effect on learners’ lives and education as it is a disorder that results from chronic distress or consecutive calamities of inefficacy in a particular profession or discipline (Salanova and Llorens, 2008).

Moreover, a significant element in deciding scholastic achievement is the capability of dealing with life stress (Kamtsios and Karagiannopoulou, 2015; Wang and Guan, 2020). To this end, various concepts have been associated with stress and burnout as the latest studies indicated that tolerance of ambiguity, as an emotional construct, may have a key role in adjusting and controlling stress (Xu and Tracey, 2014). Tolerance of ambiguity is characterized as the propensity of a person to be unable to tolerate various responses caused by the discerned lack of prominent, important, or adequate knowledge and maintained by the associated discernment of ambiguity (Carleton, 2016). A sense of ambiguity can be encountered at various phases and areas of life and language learners, for instance, may not be confident about their future objectives or career decisions. Ambiguity is overwhelming for people and can cause stress, and a deconstructive feeling, particularly for those who cannot tolerate it so those people who cannot tolerate ambiguity may regard the indispensability of life’s ambiguity as stressful (Liao and Wei, 2011).

More specifically, people who do not tolerate ambiguity experience stress and anger and believe that it is deconstructive and must be prevented, and they have difficulties operating in unpredictable conditions. Unluckily, people who do not tolerate ambiguity are intolerant of numerous circumstances, as some ambiguities happen in their daily lives and the inclination to respond reconstructively to ambiguity can result in increased stress (Buhr and Dugas, 2006). While individuals have the flexibility to respond to notions and convictions that differ from their own standpoints, others are more likely to decline notions that are inconsistent with their system and ambiguity of tolerance alludes to the way a person or group displays and handles information about an ambiguous circumstance when they experience a set of unknown, intricate, or inconsistent cues (Nezhad et al., 2013). Even though great attention is drawn to these two concepts, stress, and burnout, based on the author’s knowledge, literature on the role of tolerance of ambiguity in the language learning domain is restricted so this study attempts to inspect the EFL learners’ tolerance of ambiguity as interpreters of stress and burnout.

### Burnout

A mounting response to a persistent stressor in the workplace is known as burnout that is characterized by the symptoms of emotional fatigue, depersonalization, and poor individual achievement and student burnout seem to have a grave impact on learners’ scholastic life (Maslach and Leiter, 2016). A three-dimensional syndrome of emotional fatigue, cynicism, and expert efficacy is known as students’ burnout (Schaufeli and Taris, 2005) as academic burnout alludes to learners’ fatigue due to high learning requirements (fatigue), distant and detached behavior toward school assignments, or educators (cynicism), and a sense of incompetence or a sense of absent success as a learner (inefficacy) that is also related to the self-assessment of unsuccessful learners in the academic context (Zhang et al., 2007).

### Academic Stress

Academic stress seems to be the most regular psychological condition encountered during their coaching period (Ramli et al., 2018; Han, 2021). Stress is regularly utilized systematically to allude to a broad diversity of cycles, like life occurrences and circumstances, intellectual, emotional, and biological responses that such circumstances arouse (Epel et al., 2018). Additionally, scholastic stress is a state created by the pressure of encountering scholastic difficult circumstances among learners (Alvin, 2007). Moreover, it leads to the learners having an individual recognition of the inability to manage both environmental needs and learners’ actual assets. In fact, stress has a prolonged and uniform history as an element that adversely affects a learner’s scholastic background.

### Tolerance of Ambiguity

In a language education setting, tolerance of ambiguity is the capability of coping with new ambiguities without feeling frustration or relying on knowledge sources; thus, learners who tolerate ambiguity are anticipated to comfortably learn a new language, despite encountering uncertainties and foreign phenomena in the structural and cultural dimensions (Kamran, 2011). The absence of dependable or appropriate information is known as ambiguity that mirrors a trend of emotional responses to complexity, confounding, or unfamiliar information or occurrences (Hillen et al., 2017). Poor tolerance of ambiguity can impede the creation of social connections, performance in ambiguous environments, the attainment of intricate notions and abilities, and those who are uncomfortable with an ambiguous situation may pull out of it or keep away from the same situation in the future (Tynan, 2020). When learners face enormous amounts of new information or conflicts in their language classes, it can lead to strong negative emotional reactions such as stress. Ambiguity tolerance is generalized to various aspects of an individual’s emotional and cognitive functions and is characterized by cognitive style, belief, and viewpoint systems, associative and social functions, and problem-solving practices (Furnham and Marks, 2013). Ambiguity of tolerance is characterized as an intellectual prejudice that influences how an individual discerns, interprets, and reacts to an ambiguous circumstance at the level of intellect, emotion, and behavior (Dugas et al., 2004).

## Implications and Future Directions

Learners’ stress can lead to a loss of excitement for taking part in the scholastic study. It can also jeopardize the learner’s scholastic future. Therefore, it is crucial to find ways and determine factors that assist learners to lessen their levels of stress and burnout and it is of utmost significance to enable learners to find tactics to use in the case of challenges and to cope with them in the scholastic context, which may decrease the risk of emotional stress. This minireview provides a number of implications for learners in language learning situations. Since a high degree of stress and burnout levels are both linked to diminished life fulfillment, and critical conceptions of dropping out, these regularly bring about low performance and decreased responsibility. Based on the expanding perception of Positive Psychology in language learning (Wang et al., 2021), the concepts of stress and burnout may offer perceptions for educators in employing inhibition and involvement which are suited well into the learning situation focusing on tolerance of ambiguity as an effective way and strategies to counteract stress and burnout may be beneficial both for learners’ language progress, and their well-being. In this way, it can be argued that the intolerance of ambiguity is a person’s understanding of a circumstance centered on a person’s emotions (Dugas et al., 2004). Tolerance of ambiguity has been accepted as a significant element in the remedy of stress and burnout so types of interventions could be premeditated and employed in an educational context to expand learners’ tolerance of ambiguity to eventually diminish the level of stress and burnout (Shihata et al., 2016). Indeed, it is specifically critical to plan and employ approaches to lessen the occurrence of stress and burnout so tolerance of ambiguity, recognized as an emotional perception, can be considered as one way to deal with these problems because those learners who are exceedingly intolerant of ambiguity in case of an ambiguous incident understand the event as irritating and intolerable, while those learners who are tolerant of ambiguity consider the same phenomenon as less irritating. Learners who are more tolerant of ambiguity are more comfortable when faced with unknown cases or unpredictable in various educational circumstances. Students’ success can be affected by tolerance for ambiguity. Since everyday life is full of ambiguity, those who are intolerant of ambiguity can easily find many “reasons” to fret about (Dugas et al., 2004) and learners with more tolerance of ambiguities are more likely to apply language policies to manage and regulate ambiguities (Varasteh et al., 2016).

As a result of the negative impacts of stress and burnout on education, teaching staff representatives, counselors, and others who involve in learners could provide more control by inspiring learners to deal with the probable roots of their trauma, and by helping them find or design active and influential coping strategies to improve their intervention agendas. Furthermore, teachers are supposed to do a better job preparing and instructing their learners to contend with both their stress and consequently their burnout. In addition, more stress management training programs through workshops, seminars, and webinars should be held to assist students to be more relaxed and interested in the process of their education and arrange their future occupation policies and provide required support in their search of further instruction. As this study is a mini review and its purpose is not to implement intervention, ambiguity of tolerance could be regarded as a fundamental societal and emotive aptitude in further research that should be promoted in learners through some interventions with the purpose of addressing academic stress and burnout, so in this way, the results of such quantitative research can be added to the literature to confirm the consequences.

## Author Contributions

Both authors listed have made a substantial, direct and intellectual contribution to the work, and approved it for publication.

## Conflict of Interest

The authors declare that the research was conducted in the absence of any commercial or financial relationships that could be construed as a potential conflict of interest.

## Publisher’s Note

All claims expressed in this article are solely those of the authors and do not necessarily represent those of their affiliated organizations, or those of the publisher, the editors and the reviewers. Any product that may be evaluated in this article, or claim that may be made by its manufacturer, is not guaranteed or endorsed by the publisher.
